# Early Alterations of QTc in Patients with COVID-19 Treated with Hydroxychloroquine or Chloroquine in Libreville, Gabon

**DOI:** 10.3390/clinpract12040052

**Published:** 2022-06-30

**Authors:** Elsa Ayo Bivigou, Charlene Manomba Boulingui, Aridath Bouraima, Christian Allognon, Christelle Akagha Konde, Gabrielle Atsame, Armel Kinga, Jean B. Boguikouma, Philomène Kouna Ndouongo, Marielle K. Bouyou Akotet

**Affiliations:** 1 Cardiology Department, University Hospital of Libreville, Libreville P.O. Box 18231, Gabon; bivayo@yahoo.fr (E.A.B.); arikissb@gmail.com (A.B.); christalog4@gmail.com (C.A.); akaghakonde@yahoo.fr (C.A.K.); kinga.armel@yahoo.fr (A.K.); 2 Department of Medicine, University Hospital of Libreville, Libreville P.O. Box 18231, Gabon; manomba20@gmail.com (C.M.B.); gabrielle.atsame@gmail.com (G.A.); boguijb48@hotmail.com (J.B.B.); ndouongoph@gmail.com (P.K.N.); 3 Department of Basic Sciences, Faculty of Medicine, Université des Sciences de la Santé, Libreville BP 4009, Gabon

**Keywords:** hydroxychloroquine, chloroquine, COVID-19, QTc, Gabon

## Abstract

The objective of this study was to analyze the effect of hydroxychloroquine or chloroquine associated with azithromycin on the QTc interval in Gabonese patients treated for COVID-19. Methods: This was an observational study conducted from April to June 2020, at the Libreville University Hospital Center in Gabon. Patients admitted for COVID-19 and treated with hydroxychloroquine or chloroquine, each combined with azithromycin were included. The QTc interval was measured upon admission and 48 h after starting treatment. The primary endpoint was QTc prolongation exceeding 60 ms and/or a QTc value exceeding 500 ms at 48 h. Results: Data from 224 patients, 102 (45.5%) who received hydroxychloroquine and 122 treated with chloroquine, were analyzed. The median baseline QTc was 396 (369–419) ms. After 48 h of treatment, 50 (22.3%) patients had a significant prolongation of QTc. This tended to be more frequent in patients treated with chloroquine (*n* = 33; 27.0%) than in those treated with hydroxychloroquine (*n* = 17; 16.7%) (*p* = 0.06). QTc prolongation exceeding 60 ms was found in 48 (21.3%) patients, while 11 patients had a (4.9%) QTc exceeding 60 ms at admission and exceeding 500 ms after 48 h. Conclusion: Early QTc prolongation is frequent in COVID-19 patients treated with hydroxychloroquine or chloroquine in association with azithromycin.

## 1. Introduction

The Coronavirus disease 2019, commonly named COVID-19, is responsible for a pandemic that has affected more than 166 million people worldwide, and it is responsible for more than 3.4 million deaths, including 85,000 in Africa [1]. With 45,750 cases registered at the end of February 2022, COVID-19 prevails in Gabon with a mortality–morbidity rate comparable to that of many countries in sub-Saharan Africa (SSA) but significantly lower than that reported in developed countries [1,2]. Although many therapeutic trials have shown the lack of true efficacy of repurposed drugs for COVID-19 treatment, antimalarials and antiviral drugs are still widely used in Africa [3]. Indeed, access to the most effective therapeutics remains a challenge for SSA countries. In this context, hydroxychloroquine (HCQ) and chloroquine (CQ) are still used in several countries mainly due to their accessibility [3,4,5,6,7]. Both are aminoquinoline molecules whose antiviral action was suggested in vitro in association with azithromycin (AZN). Nevertheless, their in vivo efficacy, notably by reducing the mortality rate or as a viral cure, was not proven [8,9,10]. In Africa, the affordability and the relatively safe profile of CQ are known in the context of malaria treatment, which has justified the adoption of these molecules for COVID-19 treatment in the national recommendations of some African countries, including Gabon at the beginning of the pandemic [11]. However, HCQ and CQ are known QT prolonging drugs. The prolongation of the QTc interval is a parameter used during clinical trials to predict a treatment’s risk of arrhythmia [12,13]. It is an important precursor of wave burst arrhythmia (WBA) and potentially deadly polymorphic ventricular tachycardia (PVT) [12]. In developed countries, arrhythmia complications due to these two molecules in association with AZN in COVID-19 patients have been reported; however, data from SSA are lacking [4,5,6,7]. These data foreshadow an uncontrolled risk of the use of these molecules in the case of COVID-19. Thus, this study aims to report the early effects of HCQ or CQ in association with AZN on the QTc interval in patients treated for SARS-CoV-2 infection at the Centre Hospitalier Universitaire de Libreville (Libreville University Hospital Center, CHUL) in Gabon.

## 2. Materials and Methods

This was a prospective observational study led at the CHUL in Libreville, Gabon. It received the approval of the National Ethics Committee for Research. All patients signed an informed consent form for the administration of HCQ or CQ and the recording and the use of their data.

Data from patients hospitalized for COVID-19 from 15 April to 15 June 2020, were analyzed. Patients were selected according to the following criteria: aged over 18 years, COVID-19 infection confirmed by reverse transcription polymerase chain reaction (RT-PCR) performed in oropharyngeal or nasopharyngeal samples and treatment with HCQ or CQ. Exclusion criteria were lack of patient consent, history of quinine allergy, and uninterpretable ECG, known long QT syndrome, history of drug-induced QTc prolongation, and the presence of an acute phase of another disease.

The decision to prescribe HCQ or CQ in association with AZN for the treatment of COVID-19 infection was made by the physician in accordance with national recommendations and depending on the availability of the medications in Gabon [11]. Treatment started on the day of admission after the electrocardiogram. Indeed, at admission, there were no other antiviral drugs prescribed. The patients were admitted with a positive RT-PCR test, and they were put on treatment upon admission (the same day). The QTc were measured before the beginning of the treatment and 48 h after the first administration of HCQ or CQ. A dose of 200 mg of HCQ or 250 mg of CQ was administered twice per day for 10 days combined with two doses of 250 mg of AZN on the first day and then 250 mg per day for 4 days. Two electrocardiograms (ECGs) with 12 leads were performed: the first was performed at admission, and the second was performed 48 h after the start of the treatment. The primary endpoint was the prolongation of QTc under treatment defined by a QTc prolongation exceeding 60 ms and/or a QTc value exceeding 500 ms at 48 h. Changes in heart rhythm appearing during the treatment were also recorded.

Sociodemographic parameters (age, sex, weight, height), cardiovascular risk factors (high blood pressure, diabetes, obesity, smoking), clinical, biological (kalemia), electrocardiographic (QTc, arrhythmia and/or cardiac conduction disorders) were recorded on a case report form. Therapeutic data (use of digoxin or cordarone, discontinuation of treatment for severe ventricular arrhythmias or prolongation of QTc greater than 500 ms) were also noted.

Patient outcome (death during the 48 h of hospitalization) was also included in the analysis. Each ECG was performed using a Cardiovit-AT 101 electrocardiograph (Schiller Médical) and recorded at a speed of 25 mm/s with an amplification of 10 mm/mV. All ECGs were read by two experienced cardiologists, and all the parameters were checked twice to increase quality control. In case of discrepancy, a third independent specialist, unaware of the decision of the other, checked the ECG and the two closest interpretations were considered. The QT interval measurement was performed on the V5 or V6 Wilson leads. The QT interval was corrected (QTc) depending on the heart rate (HR) and according to Bazett’s formula (QTc = QT/√ RR) for an HR between 60 and 100 beats per minute. In the case of an HR exceeding 100 beats per minute or lower than 50 beats per minute, Fridericia’s formula was used. In the case of a bundle branch block, QTc was adjusted (adjusted QTc = QTc − QRS duration + 90 ms). The QTc on the baseline ECG was considered long for a duration exceeding 470 ms in men and 480 ms in women.

COVID-19 clinical forms were classified into four groups: pauci-symptomatic form (in the presence of flu-like syndrome and/or otorhinolaryngology signs such as anosmia or ageusia), moderate form (in the case of associated pneumonia), severe form (in the case of respiratory distress and/or uncontrolled or decompensated comorbidities), and critical form (acute respiratory distress syndrome requiring artificial ventilation and hospitalization in an intensive care unit). Obesity was defined according to the World Health Organization’s classification [14].

Parameters were entered into an Excel sheet, and statistical analysis was performed using StatView software. For quantitative variables, medians with interquartile ranges were calculated. A comparison of medians was performed with Student’s *t*-test. Qualitative variables were expressed as percentages, and they were compared with a Pearson’s chi-squared test. The significance threshold was fixed at *p* < 0.05.

## 3. Results

### 3.1. General Data

Among the 296 COVID-19 patients hospitalized at the CHUL during the study period, 72 (24.3%) were not included. These latter patients did not sign the informed consent form (*n* = 26) or had a history of allergy to quinine (*n* = 12), and they had an unusable ECG (*n* = 34). Therefore, data from 224 were analyzed.

#### 3.1.1. Sociodemographic and Clinical Data

The general characteristics of the study participants (*n* = 224) are presented in Table 1. The median age was 47.3 ± 19 years. However, 10.3% (*n* = 23) of participants were aged over 65 years. The sex ratio was 1.7. The median body mass index (BMI) was 29 (24–33) kg/m^2^. Obesity was moderate, severe, and morbid in 49 (21.9%), 22 (9.8%), and 10 (4.5%) patients, respectively. Clinically, 24 (10.7%) patients presented with a critical form of COVID-19 requiring non-invasive mechanical ventilation.

#### 3.1.2. Biological Data

Kalaemia was available at the time of the study for 183 (81.7%) participants. It was below 4.2 mEq/L, and it was found in 138 (75.4%) patients. Among the participants, 44 (24.0%) had a kalaemia value below 3.5 mEq/L, and 16 (7.1%) had a kalaemia value below 3.0 mEq/L. Hypokalaemia was found in 21.6% of patients treated with CQ and in 27.2% treated with HCQ (*p* = 0.38).

#### 3.1.3. Electrocardiogram

Sinus tachycardia (*n* = 21, 9.4%) or bradycardia (*n* = 2, 0.9%) as well as other electrocardiographic abnormalities were determined during ECG (Table 1). The median baseline QTc of the study population was 396 (369–419) ms. There was no significant difference in the median QTc according to sex: [396 (371–422) ms] in men vs. [399 (360–412) ms] in women (*p* = 0.76). Subjects aged less than 65 years had a shorter median QTc (392, 365–415 ms) vs. (408, 376–429 ms) in older patients (*p* = 0.05). Otherwise, the median QTc was comparable between diabetic [398 ms (371–426)] and non-diabetic [395 ms (371–415)] patients (*p* = 0.29). Similar trends were observed in obese patients compared to those with normal body weight. The median QTc was significantly longer in patients suffering from high blood pressure (402, 271–424 ms) than in patients without hypertension (390, 360–410 ms) (*p* = 0.02). Kalaemia did not significantly influence the median QTc, which was 397 ms (359–412) in patients with hypokalaemia and 396 ms (370–421) in patients with normal kalaemia (*p* = 0.68).

**Figure 1:**
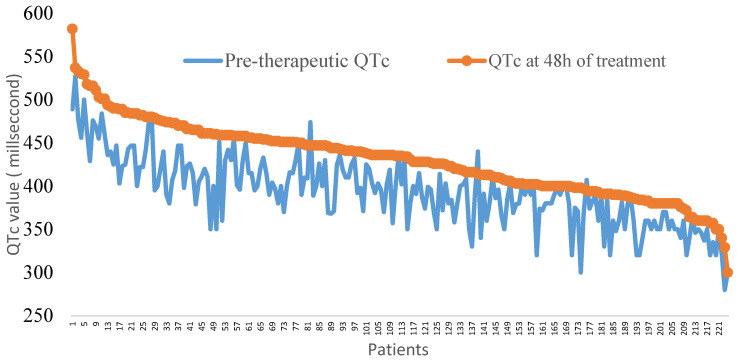
Representations of QTc values at baseline and after 48 h of treatment with hydroxychloroquine or chloroquine in 224 COVID-19 patients.

According to drug administration, patients were distributed into two groups (Figure 1). Both groups were comparable according to sex (*p* = 0.73), median age (*p* = 0.25), baseline median HR (*p* = 0.43), and distributions of cardiovascular risk factors, comorbidities, and kalaemia (*p* = 0.61) (Table 1). The median baseline QTc was longer in patients treated with CQ (400, 374–425 ms) than in those treated with HCQ (388, 360–409 ms).

### 3.2. Electrocardiographic Changes after 48 h of Treatment

The baseline QTc values and the values of patients under medication are represented in Figure 1. The median QTc and the maximum QTc were 435.0 ms (400.0–458.0) and 580 ms, respectively, for patients under treatment. QTc prolongation was not significantly different according to the type of treatment (Table 2). The maximum QTc prolongation was recorded in a patient with a baseline QTc of 350 ms and a kalaemia value of 3.66 mEq/L.

Overall, almost a quarter of the patients (22.3%, *n* = 50) had significant QTc prolongation 48 h after starting treatment. A similar proportion was found in patients with hypokalaemia (*n* = 11, 25%) and those with normal kalaemia (*n* = 30, 21.6%) (*p* = 0.63), and according to sex, 24.1% (*n* = 19) were women and 21.3% (*n* = 31) were men (*p* = 0.62). Although 30.4% (*n* = 3) of participants aged more than 65 years and 21.4% (*n* = 43) of the younger patients had QTc prolongation, the difference was not statistically significant (*p* = 0.32). Prolongations tended to be significant in diabetic patients (32.6%, *n* = 14) compared to non-diabetic patients (19.9%, (*n* = 36) (*p* = 0.07). This difference was not found in patients suffering from hypertension or obesity (*p* = 0.68). QTc prolongation exceeding 60 ms was the most frequent type of significant prolongation (Table 2). Treated patients having a QTc exceeding 500 ms were less frequent (Table 2). This alteration was significantly more frequent in diabetic patients (11.6%) than in nondiabetic patients (3.9%) (*p* = 0.04). Nine (4.2%) patients had a QTc prolongation exceeding 60 ms and 500 ms.

Considering the treatment, subjects receiving CQ tended to have QTc prolongation more frequently (*n* = 33, 27.0%) than those receiving HCQ (*n* = 17, 16.7%) (*p* = 0.06). This difference was significant when the baseline QTc was considered. In subjects treated with CQ, 83.3% had a long baseline QTc, whereas 24.1% had a normal baseline QTc (*p* < 0.01). In patients receiving HCQ, changes were not found (*p* < 0.43). Although the differential between QTc on admission and QTc at 48 h post-treatment was 8 ms higher in the case of CQ, the difference was not statistically significant. The median QTc after 48 h of treatment was significantly higher in patients who received CQ treatment than in those who received HCQ, as was the frequency of patients with a QTc > 500 ms (Table 2).

### 3.3. Arrythmia

In addition to QTc abnormalities, five (2.2%) patients who were treated developed rhythm disorders (Table 2). In patients with ventricular extrasystoles, the median kalaemia value was 3.8 (3.4–4.2) mEq/L, none had a QTc exceeding 500 ms, and three (60%) had QTc prolongation exceeding 60 ms. All patients presenting ventricular extrasystoles upon ECG were treated with CQ. Two (1.8%) participants died in intensive care after 48 h of treatment due to respiratory distress. QTc prolongation exceeding 500 ms was the only criterion selected for treatment discontinuation. The use of this sole criterion underestimated the risk of WBA in 28 patients presenting only QTc prolongation exceeding 60 ms and for whom HCQ and CQ were continued.

## 4. Discussion

The evaluation of the arrhythmogenic risk of CQ and HCQ prescribed for COVID-19 is a necessity in SSA where both molecules are still used despite reports on lack of efficacy against the virus. Although studies on the use of CQ in the context of malaria report only a few cardiac side effects [15,16], the context of SARS-CoV 2 infection seems specific. Indeed, this pathology has a different arrhythmogenic character due to a complex pathophysiology associating an important inflammatory state, hypoxia, potential myocardial injuries induced by the virus, and hydrolytic electrolytic disorders, which are frequently reported [17]. In developed countries, studies report populations at risk of developing severe forms, especially elderly individuals and those presenting with comorbidities, and possible drug interactions [4,5,6,7,9,10]. In this context, sociocultural and economic data could increase the risks, as reported by Yanci in an African American population, with a mortality rate six times higher than that in a Caucasian population [18]. Giudicessi also mentioned a different genetic susceptibility to SARS-CoV-2 infection or to therapies used in melanoderm subjects [19]. There are only a few data published in SSA to date.

In Gabon, National Guidelines for COVID-19 management included the prescription of HCQ associated with AZN at the beginning of the pandemic [11]. This work performed at the CHUL, a national reference center that cares for COVID-19 patients, reports recorded data on the evaluation of the risks linked to the prescription of these molecules. These recommendations regarding the care of COVID-19 patients stipulate that the risk of contamination of health professionals should be minimized [20]. Due to these measures, the 34 patients for whom ECG was not usable were excluded from the study since the limited staff and financial resources of the CHUL could not allow new ECG recordings.

Significant QTc prolongation was recorded in 22.3% of participants. Even though a higher prevalence was reported in other studies with rates ranging from 23% to 36%, these rates almost represent more than double the overall prevalence (10%) noted in an analysis on patients treated with HCQ or CQ and AZN, as in this work [4,5,6]. The median QTc prolongation varies depending on the study [4,5,6,7,21]. It was 33 ms at the CHUL at 48 h of treatment, whereas it varied between 16 and 35 ms in other studies [4,5,6,7,21]. Many methodological differences exist between these studies depending on the modalities of QTc interval measurements, the delay until the control measure, HCQ or CQ posology, and the association with other drugs that prolong QTc [4,5,6,7,21]. However, the two-day delay chosen for this study is much shorter than that of other studies whose maximum QTc was obtained at the end of 3.1 to 5 days [5,6,7,21,22]. The median QTc observed at the CHUL could reflect a greater sensitivity of this population to treatment. Some authors showed that cardiac arrythmias in COVID-19 patients with severe or critical disease or with myocardial impact also involve QTc prolongation, while others found that QTc prolongation is rare during COVID-19 but drugs like HCQ and azithromycin are associated with a significant risk of QTc prolongation in SARS-CoV2 infected persons; this risk increases when azithromycin is associated with HCQ [23,24,25]. Additionally, COVID-19 patients were shown to be more susceptible to drug-induced QTc prolongation [23,24,25].

However, the difference observed between HCQ and CQ treated individuals who received azithromycin, suggest a potential effect of both drugs, as observed by others. Thus, these drugs, which are used for COVID-19 with a very high dosage compared to other indications, would contribute to the QTc modification observed in treated COVID-19 patients.

Prolongation exceeding 60 ms was the main significant modification of QTc noted in 18.3% of participants. This proportion corroborates data in the literature, with rates varying between 13% and 20% according to Saleh, Mercuro, Brian, and Chorin [5,21,22]. Prolongation is a known marker of WBA risk that requires treatment discontinuation according to many recommendations, such as that of the Canadian Heart Rhythm Society [17]. This criterion had not been selected by the CHUL. QTc prolongation exceeding 500 ms is also associated with a high risk of malignant ventricular arrhythmia and sudden death [26]. This risk was reported in lower proportions (5.4%) in American and European studies, with prevalence rates ranging from 9.2% to 21% [5,6,22]. These results could be explained by the lower median baseline QTc (396 ms) of the study population compared to that of other studies whose values ranged from 435 to 455 ms [5,6,7,21].

Prolongation of the QTc interval is only one of the precursors of the risk of WBA. Thus, the degree of prolongation is not always correlated with arrhythmia [13]. Many other factors, such as genetic, clinical, biological, and therapeutic factors initiate rhythm disorders [12,13,27]. Tisdale’s risk score can be used to identify hospitalized subjects at risk of WBA [28]. However, the calculation of this score requires paraclinical parameters that are hard to access in some regions of SSA, particularly in the context of this infectious pathology. The absence of a significant association between QTc prolongation and other factors (hypokalaemia, age, sex) links these modifications to the effect of the administered treatments, with a tendency toward more important modifications on CQ. Three populations at risk were noted in this series: patients presenting with an initial long QTc, diabetic patients, and elderly subjects more than 65 years old. The prescription or continuation of a drug prolonging QT in patients with an initial long QTc, as in the case of 4% of participants in this study, requires strict ECG monitoring after 4 h of treatment, which was not possible at the CHUL [29].

Even though it was not associated with significant QTc modifications in this study, hypokalaemia is an ion disorder frequently reported during COVID-19, as in the case of 24% of patients at the CHUL and 37% in Chen’s study [30]. Several pathophysiological mechanisms have been suggested, including the role of the renin–angiotensin–aldosterone system with modification of the angiotensin clearance pathway, which fosters aldosterone synthesis [29]. Recommendations were issued, and they suggest maintaining a kalaemia value higher than 4 mEq/L [31]. The mean kalaemia value reported in this series is significantly lower than these recommendations. The use of drugs that prolong QTc in this context can be fatal [17].

No WBA was noted during the 48 h of monitoring. The low WBA prevalence reported in other studies (0.0% to 0.4%) supports the innocuous nature of these molecules in COVID-19 patients [4,5,27]. However, isolated ventricular extrasystoles in patients on CQ were reported at the CHUL. In the absence of permanent ECG monitoring, performed in telemetry in European and American studies, the absence of serious paroxysmal ventricular arrhythmias cannot be ruled out [4,5,6]. These ventricular arrhythmias led to treatment discontinuation in 42.5% of patients in intensive care units in a study performed by Bessière [4].

The main limitations of this study are the absence of telemetry, the absence of ECG data at the end of treatment, and the low proportion of patients with a severe form of COVID-19 which did not allow the analysis of QTc prolongation in relation with COVID-19 severity. This study was monocentric, thus the present results could not be generalized. The absence of a group of uninfected patients treated with CQ could also be considered as a limit, however CQ has not been widely used in Gabon for more than 20 years. Both drugs received an exceptional import license for COVID-19 treatment. Moreover, HCQ and CQ were the only molecules available during the study period. Nevertheless, this study provides data on the effects of HCQ or CQ associated with AZN in a population of African adult COVID-19 patients living in SSA. It also highlights the difficulties in monitoring these patients and the resulting risk of arrhythmia

## 5. Conclusions

The known arrhythmogenic effect of HCQ or CQ in combination with AZN would cause or increase the risk of occurrence of QTc interval prolongation in COVID-19 patients in Libreville, Gabon. Due to their lack OF clinical efficacy, the administration of these molecules must be avoided or limited to a prescription in the context of clinical trials with rigorous electrocardiographic and biological monitoring. The identification and the monitoring of subjects at risk during treatment, such as diabetic patients, elderly patients, and subjects with an initial long QTc, is necessary.

## Figures and Tables

**Table 1:** Characteristics of COVID-19 patients at the CHUL.

	Total *n* (224)	Patients Treated with HCQ *n* (102)	Patients Treated with CQ (*n* 122)	*p*
**Age (median, quartile) in years**	48 (38–56)	49 (39–57)	47 (37–55)	0.25
**Sex (*n*, %)**				
**Men**	141 (62.9)	63 (61.8)	78 (63.9)	0.73
**Women**	83 (37.0)	39 (38.2)	44 (36.0)	0.73
**FC beats/minute** **(median, interquartile)**	80.0 (69.5–90.0)	79.5 (69.0–90.0)	80.0 (70.0–90.0)	0.43
**Risk factors (*n*, %)**				
High blood pressure	74 (33.0)	35 (34.3)	39 (32.0)	0.71
Diabetes	43 (19.2)	22 (21.6)	21 (17.2)	0.41
Obesity	81 (36.2)	31 (30.3)	50 (41.0)	0.10
Tobacco consumption	8 (3.6)	3 (2.9)	5 (4.1)	0.64
**Comorbidities (*n*, %)**				
Heart failure	6 (2.7)	1(1.0)	5 (4.1)	0.15
**Clinical forms (*n*, %)**				
Asymptomatic	41 (18.3)	18 (17.6)	23 (18.8)	0.81
Moderate	141 (62.9)	69 (67.6)	72 (59.0)	0.12
Severe	18 (8.0)	6 (5.8)	12 (9.8)	0.27
Critical	24 (10.7)	9 (8.8)	15 (12.3)	0.40
**Baseline ECG abnormalities (*n*, %)**				
AF	4(1.8)	4(3.9)	0 (0.0)	0.02
Right bundle branch block	3(1.3)	1 (1.0)	2 (1.6%)	0.64
Left bundle branch block	3 (1.3)	1 (1.0)	2 (1.6%)	0.64
Repolarization disorders	24 (10.7)	11 (10.8)	13 (10.6)	0.97
First degree atrioventricular block	10 (4.5)	3 (2.9)	7 (5.7)	0.31
Left ventricular hypertrophy	53 (23.7)	28 (27.4)	25 (20.5)	0.22
Left atrial hypertrophy	54 (24.1)			
Necrosis sequel	6 (2.7)	4 (3.9)	2 (1.6)	0.23
Long QTc	9 (4.0)	6 (5.9)	3 (2.4)	0.40
**Kalaemia (median, quartile) mEq/L**	3.8 (3.4–4.2)	3.8 (3.4–4.0)	3.8 (3.5–4.2)	0.49
Treatment				
Amiodaron	4 (1.8)	4 (3.9)	0 (0.0)	0.02
Digoxin	0	-	-	-

HCQ: hydroxychloroquine; CQ: chloroquine; AF: atrial fibrillation.

**Table 2:** QTc alterations and prevalence of rhythm disorders in COVID-19 patients at 48 h.

	Total *n* (224)	Patients Treated with Hydroxychloroquine *n* (102)	Patients Treated with Chloroquine; *n* (122)	*p*
**Arrhythmias**				
Isolated ventricular extrasystoles (*n*, %)	4 (1.8)	0 (0.0)	4 (3.3)	0.06
Torsade de pointe (*n*, %)	0 (0.0)	0 (0.0)	0 (0.0)	-
Atrial fibrillation. (*n*, %)	1 (0.0)	0 (0.0)	0 (0.0)	-
**QTc alterations**				
QTc (ms) (median, interquartile)	435 (400–458)	424 (391–454)	438 (402–460)	0.02
QTc interval prolongation at 48 h (ms) (median, interquartile)	33.0 (18.5–55.0)	30.0 (19.0–53.0)	38.5 (17.0–56)	0.54
QT prolongation > 60 ms at 48 h (*n*, %)	41 (18.3)	16 (15.7)	25 (20.5)	0.35
QTc exceeding 500 ms at 48 h (*n*, %)	12 (5.3)	1 (1.0)	11 (9.0)	<0.01

## Data Availability

Not applicable.
